# Clinical and neuroendocrine correlates of childhood maltreatment history in adults with bipolar disorder

**DOI:** 10.1192/j.eurpsy.2023.621

**Published:** 2023-07-19

**Authors:** S. Donato, N. Attianese, M. Battipaglia, R. Ceres, A. M. Monteleone, G. D’Agostino, G. Cascino

**Affiliations:** ^1^University “L. Vanvitelli”, napoli; ^2^University, “Scuola Medica Salernitana”, salerno, Italy

## Abstract

**Introduction:**

Childhood maltreatment (CM) has been associated to an increased risk of developing bipolar disorder (BD). A role of the hypothalamus-pituitary-adrenal (HPA) axis in mediating trauma-related risk for adult psychopathology has been suggested but scarcely investigated in BD.

**Objectives:**

The aim of this study is to explore the impact of childhood maltreatment on clinical features of BD and on the activity of the HPA axis.

**Methods:**

One hundred and six patients participated in the study. On the basis of their history of childhood trauma, as assessed by the Childhood Trauma Questionnaire (CTQ), they were divided into a group with a history of childhood maltreatment (CM+) and a group without (CM−). Twenty-nine participants (16 with a history of childhood trauma and 13 without) underwent the cortisol awakening response (CAR) test. Saliva cortisol concentrations were determined by an enzyme immunoassay method, using a commercially available ELISA kit.

**Results:**

According to CTQ, 62 had a history of childhood maltreatment and 44 had not. CM was significantly more frequent in females than males. CM+ patients showed significant higher body mass index (p = .01), number of suicide attempts (p = .03), and more severe mania symptoms (p = .01) than CM− ones. Significant associations between lifetime suicide attempts and any type of childhood maltreatment (OR = 2.79; CI = 1.01-7.73) and between emotional abuse and the presence of psychotic symptoms (OR = 2.74, CI = 1.11-6.74) or mixed mood episodes were found (OR = 2.62, CI = 1.07-6.43). Moreover, CM+ individuals with BD exhibited a significantly reduced CAR with respect to CM− ones.

**Conclusions:**

Our results add to literature findings showing a worse clinical course in BD patients with a history of childhood maltreatment and show for the first time that childhood trauma exposure is associated to impaired CAR in adults with BD.

**Disclosure of Interest:**

None Declared

